# Supplementary material for: SDG102, a H3K36-Methyltransferase-Encoding Gene, Plays Pleiotropic Roles in Growth and Development of Maize (Zea mays L.)
Source: Int J Mol Sci. 2022 Jul 5;23(13):7458. doi: 10.3390/ijms23137458 (PMC9267571; doi:10.3390/ijms23137458)
Supplement: Supplementary file 1 [file ijms-23-07458-s001.zip › Figures S1-S6.pdf]

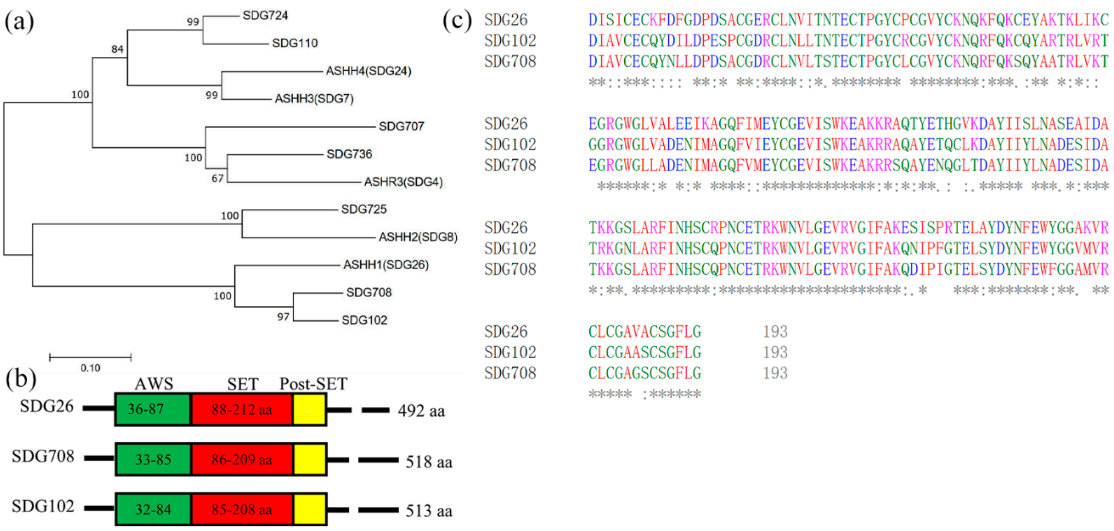

Figure S1 Phylogenetic analyses and sequence alignment of maize SDG102 to rice SDG708 and Arabidopsis SDG26. (a) Cladogram of the Ash1 SET domain proteins of maize, rice, and *Arabidopsis*. (b) Schematic diagrams of the maize SDG102, rice SDG708, and Arabidopsis SDG26. Colored boxes represent conserved protein domains: associated with SET (green, AWS); Su(var) 3-9, Enhancer of zeste, Trithorax (red, SET); and Post-SET (yellow). (c) Sequence alignment of amino acid. Identical regions are marked with asterisks (*).

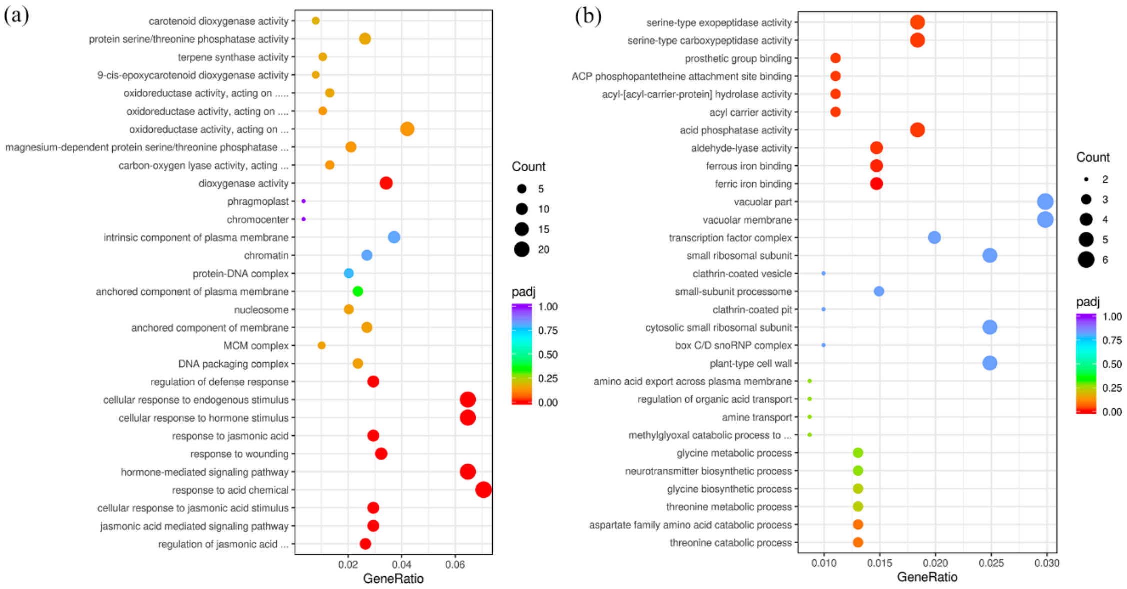

Figure S2 Differentially expressed genes (DEGs) GO enrichment analysis of AS1 and OE3. (a) Downregulated DEGs GO of AS1. (b) Upregulated DEGs GO of OE3. Adjusted P-value (padj) for each ontology term are displayed with a color reflecting its level of significance.

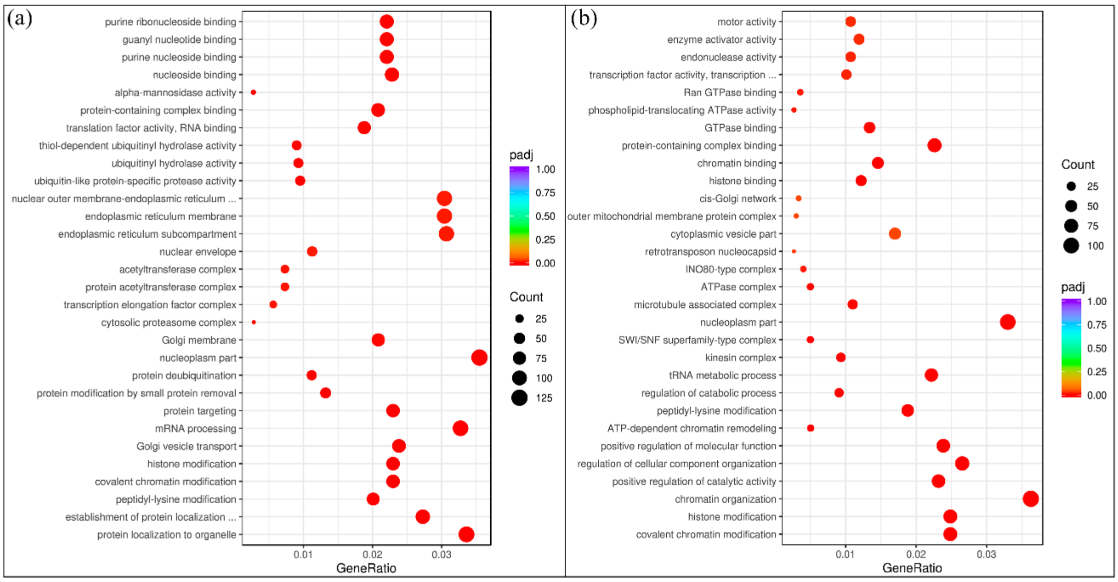

Figure S3 Significant GO terms on genes with downregulated H3K36me3 in AS1 (a) and up-regulated H3K36me3 in OE3 (b). Adjusted P-value (padj) for each ontology term are displayed with a color reflecting its level of significance.

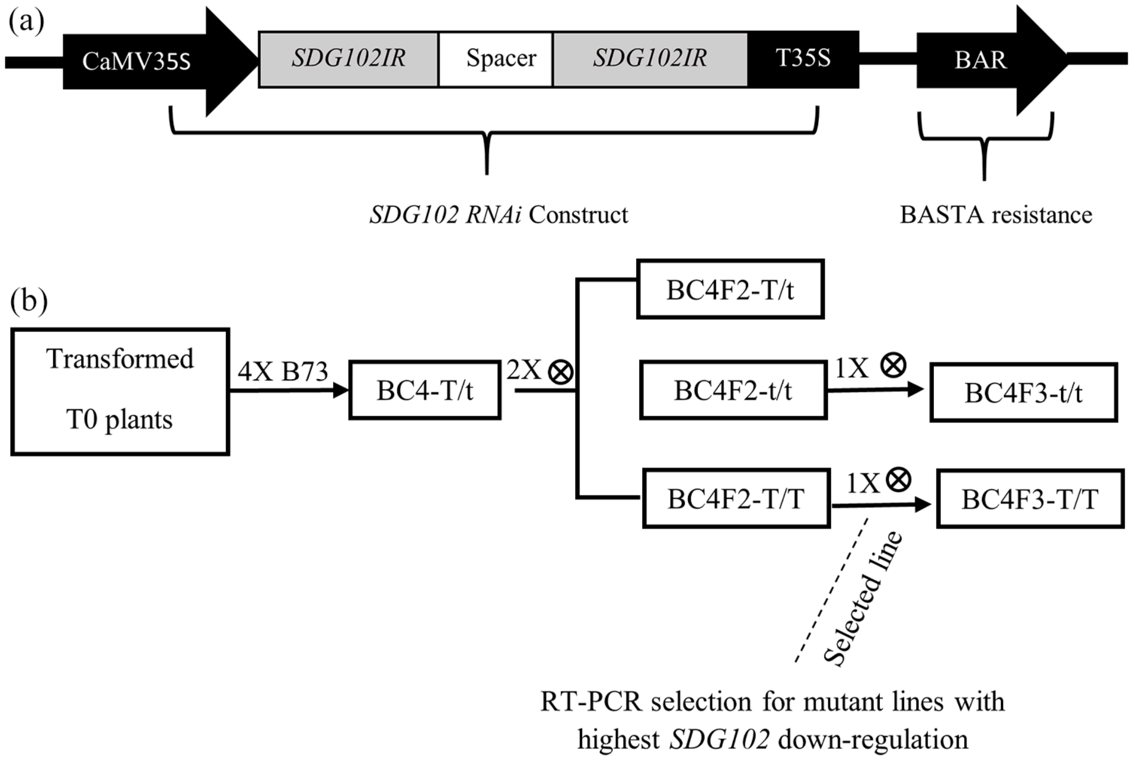

Figure S4 Schematic diagram of vector construction of *SDG102-RNAi* (a) and crossing scheme for *SDG102* down-regulation lines (b).

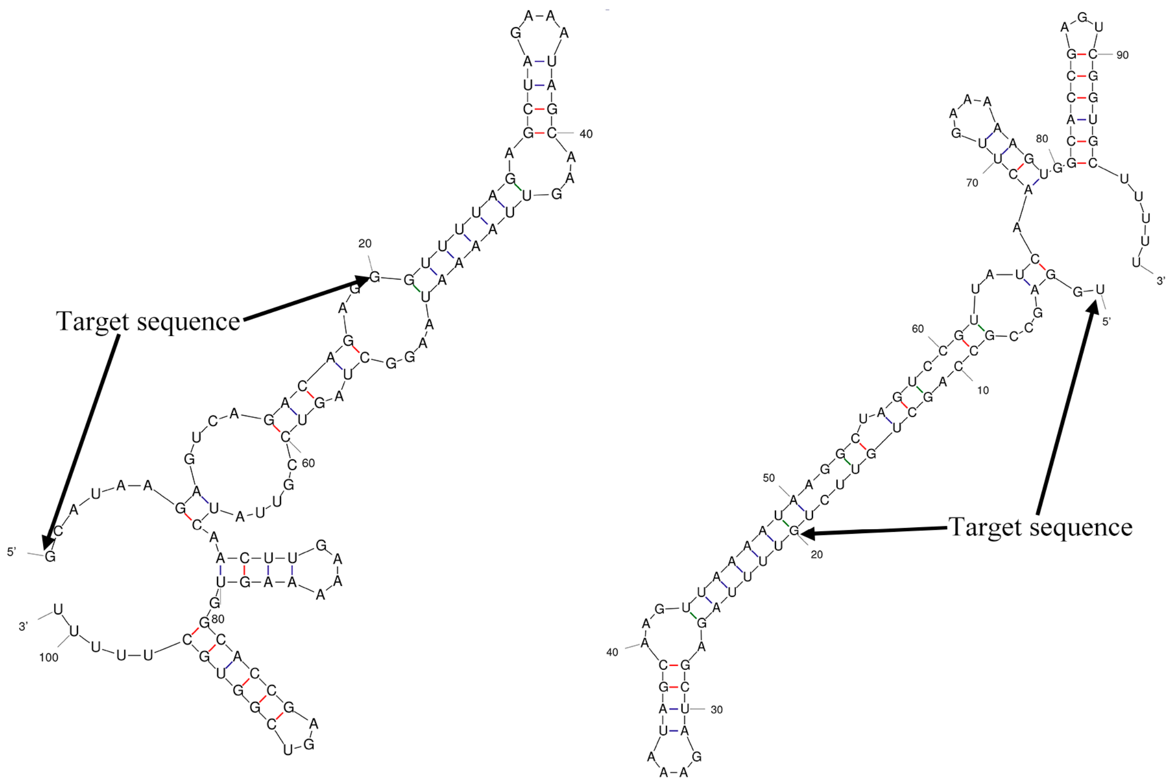

Figure S5 The secondary structures of target-sgRNAs targeting the third exon (left) and the seventh exon (right)

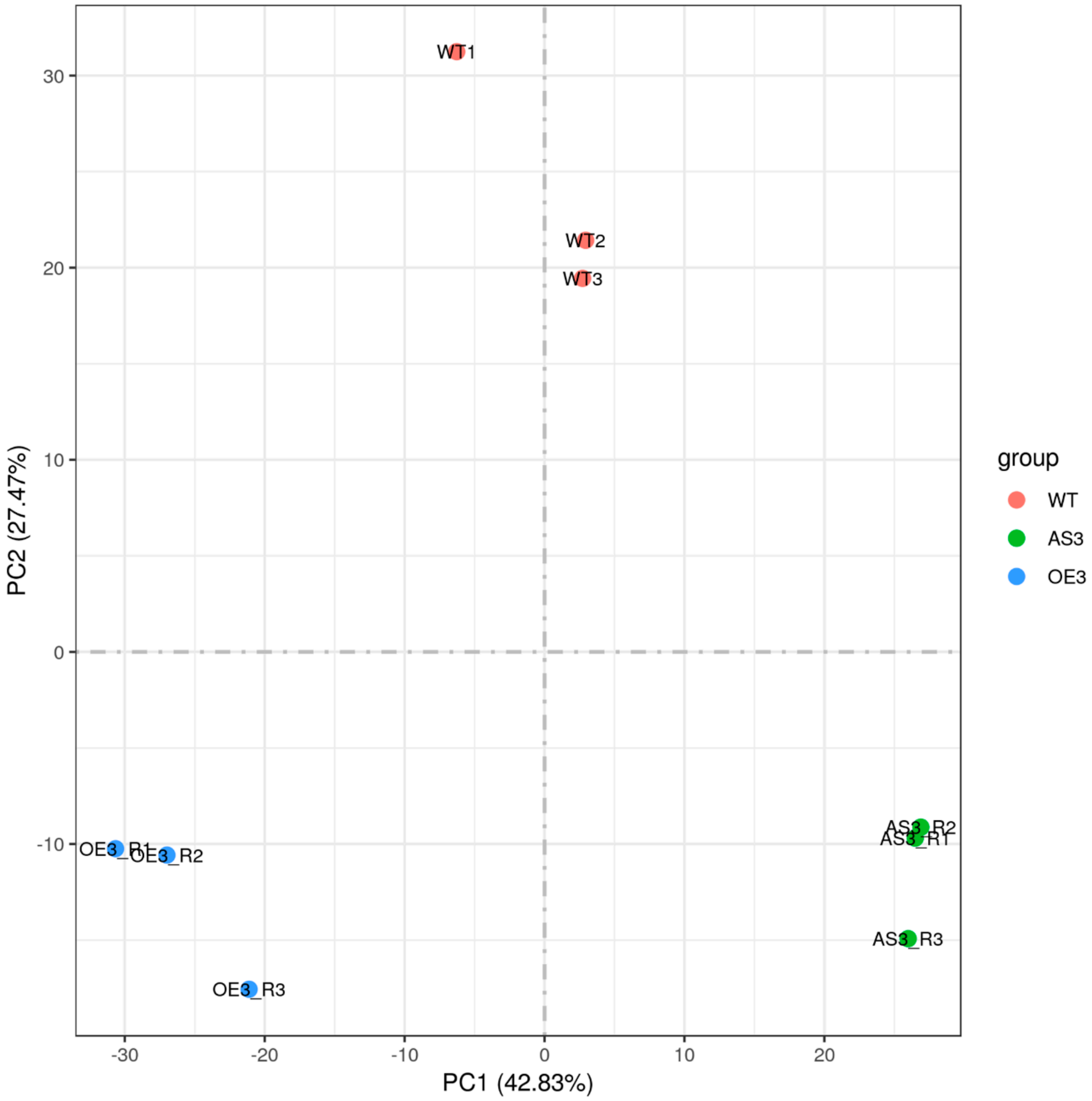

Figure S6 PCA of RNA-Seq data quality. PCA = principal component analysis
